# LAMOTRIGINE INDUCED LEUCOPENIA IN A PATIENT WITH TYPE 2 BIPOLAR DISEASE

**DOI:** 10.1192/j.eurpsy.2023.1488

**Published:** 2023-07-19

**Authors:** S. VATANSEVER, N. USTA SAGLAM, E. BESTEPE

**Affiliations:** 1 PYSCHIATRY, ERENKOY TRAINING AND RESEARCH HOSPITAL FOR PSYCHIATRY AND NEUROLOGICAL DISEASES HOSPITAL; 2 PYSCHIATRY, ISTANBUL UNIVERSITY CERRAHPASA, CERRAHPASA FACULTY OF MEDICINE, ISTANBUL, Türkiye

## Abstract

**Introduction:**

Lamotrigine(LTG) is a widely used medication for bipolar disorder(BD) maintenance treatment, bipolar depression, epilepsy, trigeminal neuralgia.^1^The well-known common side effects of LTG are rash,fatigue, gastrointestinal symptoms,dizziness,headache,insomnia.^2^While one of the most refrained side effects of LTG is Steven Johnson’s syndrome, there have been reports of blood dyscrasia,such as agranulocytosis, neutropenia, pancytopenia.^3,4^Unfortunately, the exact mechanism of the blood dyscrasias isn’t fully explained.Here we report a case of LTG-induced leucopenia in a patient with BD type 2 patient.We obtained the patient’s consent.

**Objectives:**

We report a case of a 56-year-old female patient, brought to the emergency unit with complaints of feeling unhappy, hopeless,having trouble sleeping and suicidal thoughts for two months.She attempted suicide a few days ago,had multiple suicide attempts in the last two years.She had 3 psychiatric hospitalizations due to depressive episodes and 1 hypomanic episode.Her mood was depressed.She had psychomotor retardation,no psychotic feature.Due to active suicidal ideation,we admitted her to the inpatient unit with the diagnosis of BD type 2.

Routine blood tests were within the normal range.We increased quetiapine XR 300 mg and venlafaxine 300 mg,which she had already taken;discontinued her aripiprazole treatment and added LTG 25 mg/d. 8 after initiation of LTG,there was a decrease in white blood coun(WBC) from a baseline level of 5.18x10^9^/L to 3x10^9^/L,while neutrophil count decreased from 3.8x10^9^/L to 1.15x10^9^/L in 12 days.Her medical records showed no sign of leucopenia.No pathology was detected in the peripheral smear or ultrasonography performed with the haematology consultation.Considering leucopenia might be an adverse drug reaction associated with LTG, we discontinued LTG treatment on the 9th day of administration. 9 days after discontinuation WBC was up to 4.22x10^9^/L,neutrophil count was 2.78x10^9^/L. We started valproate 500 mg/d and on the 27th day of her stay, she was discharged with a euthymic mood, having no depressive symptoms or suicidal thoughts.Her last treatment was venlafaxine 225 mg, quetiapine XR 300 mg, quetiapine IR 100 mg, valproate 500 mg, lorazepam 1 mg daily.

**Methods:**

It is a retrospective review.

**Results:**

In this LTG naive patient,the WBC values were within the normal range at admission.There was a significant temporal relationship between the initiation of the LTG and the decrease in WBC values.The absence of other factors in the laboratory tests and examinations,the rapid increase of WBC levels after the LTG was discontinued suggests the observed effect may be a side effect of LTG.

**Conclusions:**

Blood dyscrasies aren’t a very common side effect of LTG, but it might be helpful to see CBC, especially in older populations, on patients with polypharmacy regimens and with severe mental illness that may interfere with patient’s ability to express any subtle side effect.

**Disclosure of Interest:**

None Declared

